# Implementation research to improve quality of maternal and newborn health care, Malawi

**DOI:** 10.2471/BLT.16.178202

**Published:** 2017-02-22

**Authors:** Stephan Brenner, Danielle Wilhelm, Julia Lohmann, Christabel Kambala, Jobiba Chinkhumba, Adamson S Muula, Manuela De Allegri

**Affiliations:** a Institute of Public Health, Ruprecht-Karls Universität Heidelberg, Im Neuenheimer Feld 130.3, 69120 Heidelberg, Germany.; b College of Medicine, University of Malawi, Blantyre, Malawi.

## Abstract

**Objective:**

To evaluate the impact of a performance-based financing scheme on maternal and neonatal health service quality in Malawi.

**Methods:**

We conducted a non-randomized controlled before and after study to evaluate the effects of district- and facility-level performance incentives for health workers and management teams. We assessed changes in the facilities’ essential drug stocks, equipment maintenance and clinical obstetric care processes. Difference-in-difference regression models were used to analyse effects of the scheme on adherence to obstetric care treatment protocols and provision of essential drugs, supplies and equipment.

**Findings:**

We observed 33 health facilities, 23 intervention facilities and 10 control facilities and 401 pregnant women across four districts. The scheme improved the availability of both functional equipment and essential drug stocks in the intervention facilities. We observed positive effects in respect to drug procurement and clinical care activities at non-intervention facilities, likely in response to improved district management performance. Birth assistants’ adherence to clinical protocols improved across all studied facilities as district health managers supervised and coached clinical staff more actively.

**Conclusion:**

Despite nation-wide stock-outs and extreme health worker shortages, facilities in the study districts managed to improve maternal and neonatal health service quality by overcoming bottlenecks related to supply procurement, equipment maintenance and clinical performance. To strengthen and reform health management structures, performance-based financing may be a promising approach to sustainable improvements in quality of health care.

## Résumé

**Objectif:**

Évaluer l'impact, sur la qualité des services de santé maternelle et néonatale au Malawi, d'un schéma de financement fondé sur les résultats.

**Méthodes:**

Nous avons réalisé une étude contrôlée non randomisée avant/après afin d'évaluer les effets de mesures incitatives fondées sur les résultats, mises en œuvre à l'échelle des districts et des établissements de santé à destination des équipes de gestion de la santé et des agents de santé. Nous avons ainsi évalué, au sein des établissements de santé, l'évolution : des stocks de médicaments essentiels, de la maintenance des équipements ainsi que des procédures cliniques en soins obstétricaux. Des modèles de régression de l'écart des différences ont été utilisés pour analyser les effets de ce schéma sur le respect des protocoles de traitement pour les soins obstétricaux et sur la disponibilité des équipements, des fournitures et des médicaments essentiels.

**Résultats:**

Notre étude a porté sur 33 établissements de santé (23 établissements visés par le schéma d'intervention et 10 établissements témoins) pour un total de 401 femmes enceintes, dans quatre districts. Dans les établissements visés par l'intervention, ce schéma a permis d'améliorer la disponibilité des équipements fonctionnels et des stocks de médicaments essentiels. Nous avons également observé des effets positifs sur l'approvisionnement en médicaments et sur les activités de soins cliniques dans les établissements non visés par l'intervention; des effets vraisemblablement dus à l'amélioration des performances dans la gestion de la santé au niveau des districts. Le respect des protocoles cliniques par le personnel obstétrical s'est amélioré dans tous les établissements étudiés, grâce à une supervision et à un coaching plus actifs du personnel clinique par les responsables de gestion de la santé.

**Conclusion:**

Malgré un manque patent d'agents de santé et des problèmes de ruptures de stocks qui affectent l'intégralité du pays, les établissements des districts étudiés ont réussi à améliorer la qualité des services de santé maternelle et néonatale, en surmontant les difficultés liées à l'approvisionnement en fournitures, à la maintenance des équipements et aux performances cliniques. Pour renforcer et réformer les structures de gestion de la santé, le financement fondé sur les résultats pourrait constituer une approche prometteuse pour des améliorations durables de la qualité des soins de santé.

## Resumen

**Objetivo:**

Evaluar el impacto de un plan de financiación basado en el rendimiento en la calidad del servicio sanitario materno y neonatal en Malawi.

**Métodos:**

Se realizó un estudio controlado no aleatorizado del antes y el después para evaluar los efectos de los incentivos de rendimiento a nivel de distritos y de centros para trabajadores sanitarios y equipos de administración. Se evaluaron los cambios en las existencias de medicamentos esenciales, el mantenimiento del equipo y el proceso de atención obstétrica clínica en los centros. Se utilizaron modelos de regresión de diferencia en diferencia para analizar los efectos del plan sobre la adherencia a los protocolos de tratamiento de atención obstétrica y el suministro de medicamentos esenciales, suministros y equipos.

**Resultados:**

Se observaron 33 centros sanitarios, 23 centros de intervención y 10 centros de control y 401 mujeres embarazadas en cuatro distritos. El plan mejoró la disponibilidad de los equipos funcionales y de las existencias de medicamentos esenciales en los centros de intervención. Se observaron efectos positivos en cuanto a la adquisición de medicamentos y actividades de atención clínica en centros de no intervención, posiblemente en respuesta a un mejor rendimiento de la administración de distritos. La adherencia de los asistentes de parto a los protocolos clínicos mejoró en todos los centros estudiados, dado que los gestores sanitarios de los distritos supervisaron y formaron de forma más activa al personal clínico.

**Conclusión:**

A pesar de la falta de existencias en todo el país y la extrema escasez de trabajadores sanitarios, los centros de los distritos estudiados lograron mejorar la calidad del servicio sanitario materno y neonatal, pues superaron todos los obstáculos relacionados con la adquisición de productos, el mantenimiento de los equipos y el rendimiento clínico. Para fortalecer y reformar las estructuras de gestión sanitaria, una financiación basada en el rendimiento podría ser un enfoque prometedor para obtener grandes mejoras en la calidad de la atención sanitaria.

## ملخص

**الغرض:**

تقييم تأثير برنامج التمويل المعتمد على الأداء الخاص بالجودة النوعية للخدمة الصحية الموجهة للأمهات وحديثي الولادة في ملاوي.

**الطريقة:**

قمنا بإجراء دراسة مُحكمة غير عشوائية قبلية وبعدية لتقييم آثار حوافز أداء العاملين في المجال الصحي وفرق الإدارة على مستوى المقاطعات والمنشآت الطبية. وقمنا بتقييم التغييرات التي طرأت على مخزون العقاقير الضرورية في المنشآت الطبية، وصيانة المعدات، وعمليات الرعاية التوليدية السريرية. تم استخدام نماذج تحوف "الاختلافات في الفارق" لتحليل آثار البرنامج على الالتزام ببروتوكولات علاج الرعاية التوليدية، وتوفير العقاقير الضرورية والموارد والمعدات.

**النتائج:**

قمنا بملاحظة 33 **  **منشأة صحية، و23 **  **منشأة تدخل، و10 **  **منشآت مرجعية، و401 **  **امرأة حامل عبر أربع مناطق. وقد أدى البرنامج إلى تحسين توفر كلٍ من المعدات الوظيفية ومخزون العقاقير الضرورية في المنشآت الطبية التي تمت فيها عمليات التدخل. كما لاحظنا آثارًا إيجابية فيما يتعلق بشراء العقاقير وأنشطة الرعاية السريرية في المنشآت التي لم يمتد إليها التدخل، وهو ما يُعزى على الأرجح إلى تطوير أداء إدارة المنطقة. وتحسّن التزام المساعدين في عمليات التوليد بالبروتوكولات السريرية عبر جميع منشآت الدراسة مع إقدام مديري الصحة بالمنطقة إلى تقديم أعمال الإِشراف والتدريب لطواقم العمل السريري بشكل أكثر نشاطًا.

**الاستنتاج:**

على الرغم من مشكلات نقص المخزون والنقص الحاد في العاملين في المجال الصحي على مستوى البلاد، فقد تمكنت المنشآت الواقعة في منطقة الدراسة من تحسين نوعية الخدمة الصحية للأمهات وحديثي الولادة عن طريق التغلب على عوامل الاختناق المتعلقة بتوفير اللوازم، وصيانة المعدات، والأداء السريري. وحرصًا على تعزيز هياكل الإدارة الصحية وإصلاحها، فقد يمثل التمويل المعتمد على الأداء نهجًا واعدًا لإجراء تحسينات مستدامة في الجودة النوعية للرعاية الصحية.

## 摘要

**目的:**

旨在评估基于绩效的资助计划对马拉维孕产妇及新生儿卫生服务质量的影响。

**方法:**

我们采用非随机、前后对照的研究方法就地区及机构级的绩效激励对卫生工作者及管理团队的影响进行了评估。我们对机构基本药物库存、设备维护及产科临床护理流程中的变化进行了评估。我们采用回归模型双重差分析法分析了该计划对遵照产科护理治疗方案和基本药物供给、用品及设备的影响。

**结果:**

我们在四个地区观察 33 家卫生机构、23 家干预机构、10 家对照机构以及 401 名孕妇。该计划改善了干预机构中功能性设备及基本药物库存的供应情况。我们观察到在非干预机构的药物采购及临床护理活动方面产生了积极的影响，这可能是由地区管理绩效提高造成的。地区卫生管理者对临床工作者的的监督及积极指导提高了所有调查机构的助产人员对临床方案的依从性。

**结论:**

尽管在全国范围内出现缺货和卫生工作者极度短缺的现象，但是调查地区中的机构仍设法克服与用品采购、设备维修及临床表现相关的障碍，提高了孕产妇及新生儿卫生服务的质量。为了加强和改革卫生管理结构，基于绩效的资助将可能会是一种有望持续改善卫生保健的方法。

## Резюме

**Цель:**

Оценить влияние ориентированной на результат программы финансирования на качество услуг, оказываемых материнской и неонатальной службой здравоохранения в Малави.

**Методы:**

Мы провели нерандомизированное контролируемое исследование до начала и после завершения программы, чтобы оценить влияние стимулирования медицинских работников и команд управления на уровне участка и на уровне медицинского учреждения. Мы провели оценку изменений в запасах лекарственных препаратов первой необходимости, в проведении технического обслуживания оборудования и в клинических процессах акушерской помощи в медицинских учреждениях. Нами были использованы регрессионные модели «разность разностей» для анализа влияния программы на строгое соблюдение протоколов акушерской помощи и на обеспечение лекарственными препаратами первой необходимости, расходными материалами и оборудованием.

**Результаты:**

В четырех участках мы поставили под наблюдение 33 медицинских учреждения, 23 учреждения, осуществляющих вмешательство, 10 контролирующих учреждений и 401 беременную женщину. Программа повысила доступность функционального оборудования и улучшила обеспечение лекарственными препаратами первой необходимости в учреждениях, осуществляющих вмешательство. Мы наблюдали положительный эффект в снабжении лекарственными препаратами, а также в лечебной работе в учреждениях, не осуществляющих вмешательство, что, вероятно, являлось результатом повышения эффективности управления участка. Стало более строгим соблюдение клинических протоколов акушерами во всех обследованных учреждениях, поскольку руководство медицинских учреждений на участках стало более активно контролировать и обучать клинический персонал.

**Вывод:**

Несмотря на дефицит ресурсов и острую нехватку медицинских работников в национальном масштабе, медицинским учреждениям на исследуемых участках удалось улучшить качество материнского и неонатального медицинского обслуживания путем устранения острых проблем, связанных с закупками и поставками, техническим обслуживанием оборудования и клинической эффективностью. Для укрепления и реформирования структуры организации здравоохранения финансирование, ориентированное на результат, может быть перспективным подходом к устойчивому повышению качества оказания медицинских услуг.

## Introduction

Improving health-care quality is essential to health systems strengthening.^1^ Financial, material and human resource limitations challenge low- and middle-income countries to sustain satisfactory health-care quality performance.^2^ Health-care quality improvement strategies differ in focus. Some strategies focus more on health workers’ performance and the immediate micro-context in which health workers provide care. Other strategies focus more on the health system’s macro-context to address financial and fiscal constraints, inefficient resource allocations, limited donor coordination or ineffective health sector regulation.^3^^–^^5^

Performance-based financing schemes are health financing strategies that link economic aspects of health care provision (macro-context) directly to bottlenecks at the frontline of actual service delivery (micro-context) aiming to improve health-care quality.^6^^,^^7^ The schemes commonly tie payments to front-line service providers (health worker or facility) to predefined clinical and/or structural performance measures.^8^ Performance-based payments are usually managed and directly invested in further service improvements by facilities. Moving some fiscal decision-making towards front-line providers allows for additional mechanisms to overcome day-to-day limitations in service provision (such as inadequate supplies or low staff motivation) faced by health facilities.^9^^,^^10^ Many low- and middle-income countries have adopted performance-based financing schemes because of their potential to optimize both financial management and service quality (micro-context).^11^^–^^13^ The schemes may also offer more sustainability to health-care quality improvements by strengthening or redefining responsiveness and accountability across various service providers and health-system actors (macro-context).^10^^,^^14^

Failure to maintain essential service inputs, such as adequate staffing, drug procurement, equipment repair, etc. risks the overall success of health-care quality improvement programmes.^15^^,^^16^ Health-care quality depends on adequate human resource and supply chain mechanisms for procurement of drugs and supplies, particularly in systems exposed to extreme resource constraints.^17^ In many low- and middle-income countries, central-level health officials are responsible for maintaining drug stocks, essential equipment and adequate staffing across health facilities, and are thus ultimately liable for service quality at the periphery.^18^ Therefore, centrally organized health systems may benefit from strategies that introduce more decentralized structures of decision-making, responsiveness and accountability.

While performance-based financing schemes in principle are designed to help health workers and service managers to achieve better health-care quality outcomes, their actual effect in doing so remains unclear.^7^^,^^9^ Recent performance-based financing evaluations in Afghanistan^19^ and Burundi^20^ failed to detect strong effects of their schemes on health facilities’ ability to secure essential drugs and supplies. A Tanzanian scheme directly providing incentives for facilities to manage essential drugs and supplies failed to prevent stock-outs of essential equipment, medicines and commodities.^21^ The study concluded that incentives provided to facilities to improve quality-of-care performance that are tied to drug and supply management cannot overcome systemic shortcomings, such as inadequate supply chain structures.^21^ Conversely, a scheme in Zambia provided incentives to health facilities, which were tied to a programme on essential drug and supply management. The scheme enabled participating facilities to use financial rewards gained for positive performance to purchase additional drugs, which successfully reduced subsequent stock-outs.^22^ Yet generalized evidence on performance-based financing schemes’ effects on improving health-care service management processes remains limited. This is partially because many schemes narrowly focus on service utilization or the clinical aspect of health-care quality, often overlooking elements directly tied to the structural aspect.^23^

We evaluated a performance-based financing scheme in Malawi to further understand the potential of such a scheme to improve both clinical (i.e. adherence to obstetric care treatment protocols) and structural (i.e. provision of essential drugs, supplies and equipment) aspects of health-care that have an effect on providing quality care.

## Methods

### Study Setting

Malawi has a centralized tax-based health system that receives significant external donor support.^24^^,^^25^ In 2010, basic emergency obstetric care was provided free of charge by 98 (90%) public and contracted not-for-profit health facilities nationwide.^25^ Public health-care facilities receive general budget allocations from the Ministry of Health for infrastructure, equipment and salaries.^26^ District health management teams receive additional budget allocations from the Ministry of Local Government and Rural Development to procure drugs and supplies from central medical stores for all public facilities within their districts.^25^^,^^27^

Malawi’s health system faces two major challenges. First, the country has inadequate human resources and suffers from shortages of qualified health workers, poor remuneration, limited career opportunities and insufficient supervision and training opportunities.^28^^,^^29^ Second, it has a wide-spread lack of essential equipment at health centres with recurrent stock-outs of drugs and consumables.^30^^,^^31^ Consequently, public facilities struggle to provide quality health-care services.^32^ To address some of these limitations and to meet the millennium development goals 4 and 5, to reduce child and maternal mortality, the Malawian Ministry of Health introduced the Results Based Financing for Maternal and Neonatal Health (RBF4MNH) Initiative in 2013 at several emergency obstetric care facilities. Similar to other performance-based incentive schemes, the initiative provides financial rewards for maternal care providers and district health management teams upon meeting defined performance goals (performance-based incentives). It also provides conditional cash transfers (financial rewards) for pregnant women upon meeting defined compliance targets.^1^ Details about the initiative and its implementation design are presented in Box 1.

Box 1 **Malawi’s Results-Based Financing for Maternal and Neonatal Health Initiative: implementation design**

The Results-Based Financing for Maternal and Neonatal Health Initiative was introduced in Malawi in April 2013 to improve the quality of facility-based care provided to women and newborns during and within 48 hours after delivery. The initiative was implemented in selected emergency obstetric care facilities across four districts (Balaka, Dedza, Mchinji and Ntcheu). Similar to other performance-based incentive programmes, the initiative combines financial rewards for maternal care providers and district health management teams upon meeting defined performance goals (performance-based incentives) with conditional cash transfers (financial rewards) for pregnant women upon meeting defined compliance targets.^33^ Across the four districts, the initiative contracted 18 health facilities (four hospitals and 14 health centres) during phase I (April 2013–October 2014) and then expanded to include a total of 28 health facilities (5 hospitals and 23 health centres) during phase II (ongoing until 2017). Enrolment of facilities into the initiative was non-random and was based on performance of emergency obstetric care signal functions, functionality of referral system, geographical emergency obstetric care coverage and catchment population size. Each enrolled facility received initial start-up financial support conditional on need related to immediate infrastructural requirements (i.e. potable water, electricity, waste disposal, building structures, etc.). Incentives for facilities are largely tied to clinical and general service management performance; district health management team incentives are largely tied to equipment maintenance and drug supply management across all facilities within each district. At least 30% of received payments are re-invested to further improve health care quality at district and facility levels, while the remaining portion is directly shared among staff as personal bonuses.

### Study design

This paper focuses on the performance-based financing component of the initiative and assesses its effects on maternal and newborn health service quality. The study is part of a larger impact evaluation that uses a non-randomized controlled before and after study design with independent controls.^34^ The intervention sample for this study initially consisted of all 18 facilities contracted by the initiative at the beginning of phase I: four public district hospitals, and 14 public health centres providing at least basic emergency obstetric care. The control sample included all health facilities within the same study districts that were not, or not yet, enrolled in the initiative at baseline: one mission hospital, two private and 12 public health centres providing basic emergency obstetric care. Several months prior to the last data collection round, five previous control facilities (a mission hospital, one private and three public health centres) were contracted by the initiative (phase II) and thus moved into the study’s intervention arm. Data were collected at three time-points: before the initiative was implemented (March/April 2013) used as baseline, at mid-point (June/July 2014) and at the end (June/July 2015). In addition, at each time point we identified a small number of pregnant women giving birth per each sampled facility using a convenience-sampling approach.

### Outcome indicators

We measured the scheme’s effect on quality using two sets of performance outcomes: structural performance and clinical performance (Box 2). For structural performance, we assessed the availability and functionality of service input elements, such as equipment, drug and supply items included in the initiative’s tracer lists at each sampled facility. To measure effects on clinical performance, we observed the following processes: (i) routine care activities required to identify respective emergency conditions; (ii) the overall number of pregnant women monitored by partograph; (iii) the completeness of partograph documentation; (iv) a set of common routine infection prevention measures; (v) oxytocin administration; and (vi) other essential elements of active management of third stage labour (AMTSL), such as controlled cord traction, uterine massage, completeness check of placenta and estimation of overall blood loss.

Box 2 **Performance indicators and related measures of the performance-based financing scheme in Malawi**

**Clinical performance indicators and process measures^a^**

A. Proportion of pregnant women with unknown HIV status giving birth in the facility who are tested and treated (if positive) with PMTCT medicines.

1. Birth attendant verifies patient’s HIV status using antenatal history review.

B. Proportion of pregnant women giving birth in the facility and show signs of pre-eclampsia or eclampsia who received magnesium-sulfate.

1. Birth attendant asks patient for symptoms related to pre-eclampsia.

2. Birth attendant checks for signs of pre-eclampsia.

C. Proportion of partographs completed and appropriately filled among women giving birth in the facility.

1. Birth attendant monitors partograph during first stage labour

2. Birth attendant documents record of complete partograph.

D. Implementation of at least one infection control action per reporting period.

1. Birth attendant performs hand hygiene before patient contact.

2. Birth attendant correctly uses sterile gloves during vaginal examination and stage 2 labour.

3. Birth attendant decontaminates perineum before vaginal examination and stage 2 labour.

4. Birth equipment is set up in sterile manner before the beginning of stage 2 labour.

E. Proportion of pregnant women giving birth in the facility who receive oxytocin in third stage of labour.

1. Birth attendant performs medical AMTSL.

2. Birth attendant performs practical AMTSL.

3. Birth attendant confirms success of AMTSL.

**Structural performance indicators and input measures^b^**

A. Proportion of facilities with functional operational maternity equipment.

1. Availability of routine equipment (delivery kits, suturing kits, cord clamps, manual suction device with tubing).

2. Availability of vital sign assessment equipment (blood pressure machine, stethoscope, thermometer, fetoscope).

3. Availability of emergency equipment (newborn size bag valve with mask, vacuum extractor).

4. Availability of ancillary equipment (sterilizer, number of delivery beds, number of obstetric examination beds).

B. Proportion of at least a 1-month supply of essential maternal and newborn health medicines and commodities at all facilities in the district.

1. Proportion of facilities with stocks of antibiotics (ampicillin, benzyl penicillin, co-trimoxazole).

2. Proportion of facilities with stocks of emergency obstetric care drugs (magnesium sulfate, diazepam, oxytocin).

3. Proportion of facilities with stocks of isotonic fluids (Ringer’s lactate, normal saline, dextrose 5% saline).

4. Proportion of facilities with stocks of pain control (paracetamol, pethidine).

5. Proportion of facilities with stocks of PMTCT drugs (nevirapine syrup, ART regimen 5A^c^).

6. Proportion of facilities with stocks of ancillary items (urine protein testing strips).

AMTSL: active management of third stage labour; ART: anti-retroviral treatment; HIV: human immunodeficiency virus; PMTCT: prevention of mother-to-child transmission of HIV.^a^ At birth attendant level.^b^ At facility level.^C^ Antiretroviral therapy with tenofovir, disoproxil, fumarate, lamivudine and efavirenz.Note: Based on indicators for the Results-Based Financing for Maternal and Neonatal Health Initiative.

### Data collection

We measured changes in the outcome variables (Box 2) over the study period using two different survey instruments: (i) facility inventory checklists to collect information on structural performance; and (ii) an observation checklist to document non-participant observations of routine labour cases to assess birth attendants’ clinical performance.^35^ We trained research assistants with a background in midwifery to observe and record routine clinical care processes in uncomplicated labour presenting to a facility during a 5-day visit, long enough to observe an adequate number of deliveries. We observed only for 3 days during baseline data collection. Clinical performance was measured against national midwifery standards.^36^ All uncomplicated labour case observations began when a woman in labour was taken to a maternity unit and ended two hours after childbirth. To ensure data completeness and accuracy, checklists were designed to prompt research assistants to complete or indicate any missing information during and immediately after data collection. Written informed consent was obtained from both patients and birth attendants before observation. Both survey instruments were used at all study facilities during each of the three data collection time points. We observed 33 health facilities (23 intervention facilities and 10 control facilities) and 401 pregnant women across four districts. Table 1 presents a summary of the number of sampled facilities and pregnant women observed at each data collection time.

**Table 1:** Sample size for intervention and control facilities, by data collection time point, Malawi, 2013–2015

Data collection time point	Facility type, no.		
	Intervention^a^	Control	All
**Facilities**	** **	** **	** **
Baseline	17	14	31
Mid-term	18	13	31
End-term	23	10	33
**Pregnant women**			
Baseline	63	24	87
Mid-term	106	58	164
End-term	131	19	150

^a^ The intervention was performance-based financing, a scheme where financial rewards for maternal care providers and district health management teams were provided upon meeting defined performance goals (performance-based incentives), and conditional cash transfers (financial rewards) were provided for pregnant women, upon meeting defined compliance.

### Analytical approach

To estimate the scheme’s effect on each outcome variable, we used a difference-in-differences (DiD) linear regression model.^37^

The model is as follows:

Y_i_ = β_0_ + β_1_ t1_i_ + β_2_ T1_i_ + β_3_ t1_i_*T1_i_ + β_4_ t2_i_ + β_5_ T2_i_ + β_6_ t2_i_*T2_i_ + β_k_ X_k,i_ + ρ_m,c_ + ε_it_

where, *Y_i_* represents our outcome variables, (structural or clinical performance); *β_0_* is the average frequency of observations among controls at baseline; *t* is a dummy variable indicating the observation time point, with *t1* being observation at mid-term and *t2* being observation at end-term; *T* is another dummy variable that indicates the treatment time point, with *T1* being treatment distribution during phase I (April 2013–October 2014) and *T2* being treatment distribution at phase II (from November 2014 onwards). T*t denotes the interaction between treatment and time point, where T1*t1 denotes interaction at mid-term and T2*t2 at end-term; *X_k_,_i_* denotes potential confounders; *ρ* is within-cluster correlation; *m* is the number of clusters and c denotes the number of individual observations. The overall effect size estimate at end-term is represented by *β_6_*, which served as overall effect estimate for the entire initiative.

We adjusted all models for potential confounders, i.e. facility type (hospital versus health centre) and ownership (public versus faith-based). Models used in analysing clinical performance were further adjusted for duration of stage 1 labour (accounting for late presenting women unlikely to be monitored by partograph), number of birth attendants available during a case (accounting for workload constraints) and birth attendants’ participation in in-service trainings (accounting for recent skill changes independent of the scheme). We further controlled clinical performance for clustering effects at the level of the facility. Given the relatively small sample sizes, we used bootstrapping to derive more robust estimates of the underlying population parameters.^38^ We used Stata version 12.1 (StataCorp. LP, College Station, United States of America) for all statistical analyses.

Ethical approval was obtained from the College of Medicine Research and Ethics Committee at the University of Malawi and the Ethical Committee of the Medical Faculty at the University of Heidelberg.

## Results

Table 2 summarizes changes and effect sizes for equipment maintenance. Positive DiD coefficients indicate a comparatively larger increase in availability of operational equipment at intervention facilities. We found statistically significant positive effects for nearly all essential equipment items related to routine obstetric care and vital sign recording. For items related to emergency and ancillary equipment, only a few items showed a significant positive effect (vacuum extractors, sterilizers and examination beds). We observed no significant effects for delivery beds. For blood pressure machines and newborn resuscitation equipment, improvements in both intervention and control facilities over time led to no significant change.

**Table 2:** Impact of performance-based financing scheme on equipment maintenance, Malawi, 2013–2015

Observed outcome variable by facility type	Health facilities with equipment item available in operational state at time of visit			Overall effect size	
	Baseline No.^a^ (%)	Mid-term No.^a^ (%)	End-term No.^a^ (%)	DiD (% point)	*P*
**Routine obstetric care equipment**					
Delivery kits					
Intervention^b^	10 (60)	17 (97)	20 (87)	29	< 0.01
Control	8 (60)	8 (59)	6 (58)		
Suturing kits					
Intervention^b^	1 (7)	11 (61)	13 (55)	49	< 0.01
Control	3 (25)	3 (2)	4 (45)		
Cord clamps					
Intervention^b^	8 (48)	9 (49)	14 (60)	24	0.05
Control	8 (58)	6 (48)	5 (45)		
Manual suction device					
Intervention^b^	17 (9)	4 (23)	13 (55)	43	< 0.01
Control	4 (25)	0 (0)	2 (21)		
**Vital sign recording equipment**					
Blood pressure machine					
Intervention^b^	11 (62)	14 (75)	18 (80)	−2	0.85
Control	6 (44)	7 (55)	6 (64)		
Stethoscope					
Intervention^b^	6 (34)	14 (75)	20 (88)	52	< 0.01
Control	10 (71)	9 (69)	8 (75)		
Thermometer					
Intervention^b^	12 (68)	14 (75)	22 (98)	19	0.03
Control	10 (74)	9 (70)	9 (87)		
Fetoscope					
Intervention^b^	11 (63)	8 (42)	16 (70)	19	0.09
Control	14 (99)	5 (42)	9 (86)		
**Emergency obstetric care equipment**					
Newborn size bag valve mask					
Intervention^b^	11 (67)	4 (21)	17 (75)	−6	0.58
Control	8 (56)	0 (1)	7 (71)		
Vacuum extractor					
Intervention^b^	9 (53)	10 (55)	19 (82)	43	< 0.01
Control	8 (58)	2 (14)	4 (43)		
**Ancillary equipment**					
Sterilizer					
Intervention^b^	9 (55)	13 (73)	17 (75)	27	0.02
Control	11 (77)	7 (53)	7 (69)		
At least 2 delivery beds					
Intervention^b^	19 (87)	16 (90)	20 (87)	−6	0.51
Control	11 (81)	10 (77)	9 (87)		
At least 1 obstetric examination bed					
Intervention^b^	6 (34)	17 (93)	22 (96)	33	0.01
Control	6 (44)	10 (79)	7 (73)		

DiD: difference-in-differences estimate.

^a^ Frequencies are estimates based on the regression analysis.

^b^ The intervention was performance-based financing, a scheme where financial rewards for maternal care providers and district health management teams were provided upon meeting defined performance goals (performance-based incentives), and conditional cash transfers (financial rewards) were provided for pregnant women, upon meeting defined compliance.

Note: DiD estimates are calculated across years 1 and 2.

The scheme’s effects on drug and consumable stocks varied greatly (Table 3). We observed statistically significant positive effects for ampicillin, oxytocin, dextrose 5% saline solution, pethidine, and human immunodeficiency (HIV) drugs used for prevention of mother-to-child transmission. We observed significant negative effects for benzyl penicillin, normal saline and Ringer’s solution. In both intervention and control facilities, diazepam and urine testing strip stocks improved but stocks for magnesium sulfate worsened.

**Table 3:** Impact of performance-based financing on availability of essential drug and consumables stocks, Malawi, 2013–2015

Observed outcome variable by facility type	Health facilities with item in stock at time of visit			Overall effect size	
	Baseline, No.^a^ (%)	Mid-term, No.^a^ (%)	End-term, No.^a^ (%)	DiD, (% point)	*P*
**Antibiotics **					
Ampicillin					
Intervention^b^	0 (0)	0 (0)	6 (26)	47	< 0.01
Control	3 (22)	0 (0)	2 (15)		
Benzyl penicillin					
Intervention^b^	13 (74)	17 (94)	11 (47)	−20	0.07
Control	12 (86)	9 (70)	8 (75)		
Co-trimoxazole					
Intervention^b^	15 (86)	16 (86)	20 (89)	−7	0.46
Control	11 (79)	10 (73)	9 (89)		
**Emergency drugs **					
Magnesium sulfate					
Intervention^b^	16 (92)	13 (71)	17 (75)	9	0.52
Control	10 (71)	8 (63)	5 (46)		
Diazepam					
Intervention^b^	7 (43)	7 (39)	15 (63)	−10	0.51
Control	5 (37)	4 (28)	7 (67)		
Oxytocin					
Intervention^b^	17 (99)	10 (57)	23 (100)	31	< 0.01
Control	11 (77)	7 (56)	5 (51)		
**Emergency obstetric care equipment **					
Ringer’s lactate					
Intervention^b^	8 (50)	12 (65)	14 (61)	−26	0.03
Control	3 (21)	5 (36)	6 (61)		
Normal saline					
Intervention^b^	12 (69)	16 (87)	17 (72)	−30	< 0.01
Control	6 (41)	8 (60)	7 (74)		
Dextrose 5% saline					
Intervention^b^	10 (56)	15 (84)	23 (100)	38	< 0.01
Control	9 (66)	10 (78)	7 (71)		
**Pain/fever control**					
Paracetamol					
Intervention^b^	13 (74)	16 (89)	16 (72)	4	0.74
Control	12 (84)	9 (69)	8 (78)		
Pethidine					
Intervention^b^	1 (3)	0 (0)	2 (11)	9	0.03
Control	0 (1)	1 (8)	1 (1)		
**PMTCT drugs**					
Nevirapine syrup					
Intervention^b^	12 (68)	15 (82)	20 (86)	24	0.03
Control	12 (84)	7 (57)	7 (72)		
ART regimen 5A					
Intervention^b^	16 (69)	4 (22)	22 (95)	39	< 0.01
Control	11 (80)	3 (27)	7 (67)		
**Ancillary**					
Urine protein testing strips					
Intervention^b^	10 (30)	2 (12)	9 (38)	2	0.63
Control	1 (6)	3 (25)	1 (12)		

ART: anti-retroviral treatment; DiD: difference-in-differences estimate; HIV: human immunodeficiency virus; PMTCT: prevention of mother-to-child transmission of HIV.

^a^ Frequencies are estimates based on regression analysis.

^b^ The intervention was performance-based financing, a scheme where financial rewards for maternal care providers and district health management teams were provided upon meeting defined performance goals (performance-based incentives) with conditional cash transfers (financial rewards) for pregnant women, upon meeting defined compliance.

Note: DiD estimates are calculated across years 1 and 2.

The effects on clinical performance were diverse (Table 4). Positive, but non-significant effects were observed for review of pre-eclampsia symptoms and timely set-up of sterile birth equipment. Negative effects were found for partograph monitoring and AMTSL. Initially, parallel improvements across intervention and control facilities occurred for examination of pre-eclampsia signs, partograph documentation, infection prevention techniques during vaginal examinations and births, as well as medical and practical elements of AMTSL.

**Table 4:** Impact of performance based financing on clinical performance of birth attendants, Malawi, 2013–2015

Observed outcome variable by facility type	Labour cases with clinical performance observed			Overall effect size	
	Baseline No.^a^ (%)	Mid-term No.^a ^(%)	End-term No.^a^ (%)	DiD (% point)	*P*
**Routine obstetric care**					
Patient’s HIV status checked or reviewed					
Intervention^b^	46 (73)	65 (61)	77 (59)	−4	0.82
Control	20 (81)	36 (63)	15 (81)		
Patient asked for pre-eclampsia symptoms^c^					
Intervention^b^	13 (20)	59 (56)	130 (99)	65	0.12
Control	17 (71)	26 (45)	16 (86)		
Patient is checked for pre-eclampsia signs^d^					
Intervention^b^	39 (62)	106 (100)	88 (67)	−1	0.98
Control	24 (100)	58 (100)	19 (100)		
**Monitoring of labour progression during stage 1**					
Patient monitored by partograph					
Intervention^b^	42 (66)	65 (61)	80 (61)	−24	0.14
Control	15 (64)	36 (63)	16 (82)		
Complete partograph documentation^e^ on monitored cases					
Intervention^b^	11 (17)	54 (51)	54 (41)	3	0.86
Control	1 (6)	7 (11)	5 (28)		
**Infection prevention**					
Hand hygiene before each patient contact^f^					
Intervention^b^	18 (28)	34 (32)	49 (37)	12	0.59
Control	5 (22)	15 (26)	4 (20)		
Sterile glove use during vaginal exam and stage 2					
Intervention^b^	34 (53)	67 (63)	95 (72)	12	0.61
Control	13 (56)	38 (65)	12 (63)		
Decontamination of perineum before vaginal exam and stage 2					
Intervention^b^	21 (33)	34 (32)	66 (51)	8	0.71
Control	5 (20)	12 (21)	6 (29)		
Birth equipment set up in sterile manner before stage 2 begins^g^					
Intervention^b^	43 (68)	77 (73)	114 (87)	21	0.11
Control	19 (79)	44 (76)	15 (78)		
**AMTSL**					
Medical management of stage 3^h^					
Intervention^b^	58 (92)	100 (94)	131 (100)	−18	0.17
Control	16 (68)	51 (87)	18 (94)		
Practical management of stage 3^i^					
Intervention^b^	45 (72)	83 (78)	112 (85)	−24	0.16
Control	11 (44)	43 (74)	16 (83)		
Confirmatory management of stage 3^j^					
Intervention^b^	41 (65)	42 (40)	91 (70)	−46	0.01
Control	5 (19)	25 (44)	13 (70)		

AMTSL: active management of third stage labour; DiD: difference-in-differences estimate; HIV: human immunodeficiency virus.

^a^ Frequencies are estimates based on regression analysis.

^b^ The intervention was performance-based financing, a scheme where financial rewards for maternal care providers and district health management teams were provided upon meeting defined performance goals (performance-based incentives) with conditional cash transfers (financial rewards) for pregnant women, upon meeting defined compliance.

^c^ Recent history of headache, blurriness, convulsions, pregnancy-induced hypertension.

^d^ Blood pressure check, check for swelling or oedema.

^e^ Defined as documentation of fetal heart rate every 30 minutes, uterine contractions/maternal pulse/maternal blood pressure/fetal descent every 60 minutes.

^f^ Defined as washing of hands with water and soap.

^g^ Includes setting up sterile delivery kit contents, sterile cord clamps, sterile gloves.

^h^ Parenteral administration of oxytocin once stage 3 is entered.

^i^ Controlled cord traction and uterine massage to actively support delivery of placenta.

^j^ Placenta examined for completeness and estimation of overall blood loss during delivery to determine woman’s risk of bleeding.

Note: DiD estimates are calculated across years 1 and 2 while controlling for duration of stage 1 labour, number of birth attendants available during a case, number of birth attendants’ participation in service trainings, as well as clustering effects at the level of the facility.

## Discussion

Our findings demonstrate that performance incentives directed to a range of health system actors beyond the actual frontline providers can affect health service quality positively. In our study, direct district level involvement not only improved supply management at intervention facilities, but likely produced additional benefits to control facilities.

Our findings indicate an overall positive impact on both equipment maintenance and drug stocks as a result of the incentives provided through the scheme. Though limited, this evidence suggests that performance-based financing may effectively sustain service inputs and processes once incentives are set across relevant health system levels. Qualitative evidence from interviews with the initiative’s district health and facility managers illustrate that local adoption has led to further decision-making by managers about resource allocation to extend beyond the initiative.^39^ The decision allowed facilities not included in the performance-based financing initiative to receive procurement support.^39^ This local adoption effect on equipment availability was also demonstrated by the Salud Mesoamérica initiative – a results-based funding programme that provides incentives to central health system levels to indirectly support front-line service providers.^40^^,^^41^

However, in our study, for some items availability did not improve. The number of delivery beds, for instance, remained unaffected, probably because most facilities counted at least two delivery beds already at baseline. Some drugs also remained unchanged (co-trimoxazole, paracetamol) or declined (benzyl penicillin, magnesium sulfate, isotonic fluids), either indicating increased use (e.g. in case of the incentivized administration of magnesium sulfate) or worsening central drug shortages. A randomized-controlled trial from the Democratic Republic of the Congo comparing performance-based versus fixed payments observed significant decline in essential equipment availability at facilities supported by a performance-based financing scheme.^42^ However, in this scheme, incentives were provided directly to the facility level without including district or central level entities.

In our study, Malawi’s nationwide economic situation deteriorated before our mid-term data collection which may have had an effect on drug and consumable stocks. Mixed or inconclusive effects were also reported from performance-based financing experimental work in the Democratic Republic of the Congo^42^ where after the implementation of the scheme, drug stock-outs decreased, though stock-outs of vaccines increased. Findings like these point towards overall political or economic challenges affecting health system and performance-based financing scheme operations. In contexts where availability of medical supplies is negatively affected by performance-based financing schemes, a detailed investigation may provide further understanding of system-wide determinants of performance-based financing implementation.

Among those essential drugs whose observed availability was not strongly affected by the initiative, many (except penicillin and Ringer’s lactate) nevertheless remained in stock in more than 70% of intervention facilities at end-term, which may reflect facilities’ new financial autonomy, such as using reward earnings to purchase additional drugs from private pharmacies. This aligns with findings from another performance-based financing evaluation in the Democratic Republic of the Congo, where facility managers with financial autonomy to use performance rewards to purchase drugs and supplies made decisions resulting in significant improvements in drug availability.^43^ In our study, as most of these less available drugs are essential to the medical management of obstetric complications (i.e. broad-spectrum antibiotics, crystalloid fluids, anticonvulsants), some shifts in facility stocks towards second-line tracer drugs (i.e. ampicillin, dextrose 5% saline, diazepam) occurred. These shifts likely indicate better strategic procurement decisions by district and facility managers in response to variations in first-line drug availability at central stores.

Improved availability of some items (e.g. blood pressure machines, newborn resuscitation equipment, obstetric examination beds, diazepam, Ringer’s solution) was also observed in control facilities. District-level performance indicators were purposefully designed to introduce spillover benefits to those facilities not yet enrolled. Improved equipment and supply availability in control facilities may represent such programme-induced spillover. In addition, qualitative evidence from interviews with the initiative’s health managers in the study districts demonstrate that the direct involvement of the district health management team led to more frequent performance review meetings at district levels and closer performance supervision across facilities.^39^

Our findings show few positive effects on clinical processes due to the scheme. For pre-eclampsia assessment of risk factors, we only observed improvements in symptom review, while physical examination of clinical signs increased only during mid-term data collection. There was also no effect observed on the routine review of patients’ HIV status. Mixed effect patterns regarding clinical assessment practices in maternal and child services, both history- and exam-based, were also observed in performance-based financing schemes in Afghanistan and Egypt.^19^^,^^44^ Although less obvious, these findings may nevertheless point towards the potential the schemes bring to improve providers’ adherence to routine care standards and clinical guidelines.

In respect to infection prevention processes, we observed non-significant positive effects on sterile set-up of birth equipment, but no effects on hand washing, use of gloves and perineal cleansing. This may reflect a complementary effect regarding infection prevention processes between clinical performance and traced supply incentives, which included sterile delivery packs and functional sterilizers, but not gloves, topical antiseptic solutions or other consumables relevant to infection prevention. The role of complementary use of incentives may also explain findings in a study from Afghanistan,^19^ which failed to detect significant effects on universal precautions in the absence of relevant equipment and supply improvements.

We further observed significant negative effects on processes related to partograph monitoring and AMTSL. As oxytocin (tracer item) and partograph (not listed as tracer item, but followed by the study’s facility inventory) availability significantly improved in intervention facilities, stock-outs don’t seem to be a likely cause of the negative effects. The negative trends for AMTSL performance might rather relate to district level spillover benefits to control facility performance as indicated by qualitative evidence outlining how district managers reinforced clinical standards, supervision and staff coaching across all facilities.^39^ In the case of AMTSL, control facilities – starting at a much lower performance level, but rapidly catching up over time – probably benefitted more than facilities in the intervention arm. A recent study from Burundi found that introduction of a performance-based financing scheme encourages guideline adherence through increased supervisory, coaching and technical support by senior staff.^45^ Regarding labour monitoring, birth attendants at both intervention and control facilities improved partograph documentation, a relatively time-consuming process. Yet, the proportion of overall partograph-monitored cases stagnated in the intervention facilities, which may simply reflect capacity limitations due to staffing constraints once service utilization at intervention facilities increased.

Our study has some methodological limitations. As with other studies on quality of clinical care, our sample sizes for both facilities and observed cases were relatively small therefore our study lacked the statistical power to detect additional effects that may have been produced by the scheme. Also, due to logistical and cost considerations, our evaluation design had to rely on control facilities within intervention districts, falling short in conceptually isolating district-level from facility-level effects. Our study therefore was not able to discern whether improvements in control facilities represent district-level induced spillover or rather resulted from other secular trends.
